# Electrical aspects of skin as a pathway to engineering skin devices

**DOI:** 10.1063/5.0064529

**Published:** 2021-11-18

**Authors:** Yuina Abe, Matsuhiko Nishizawa

**Affiliations:** Department of Finemechanics, Graduate School of Engineering, Tohoku University, 6-6-01 Aramaki-aza Aoba, Aoba-ku, Sendai 980-8579, Japan

## Abstract

Skin is one of the indispensable organs for life. The epidermis at the outermost surface provides a permeability barrier to infectious agents, chemicals, and excessive loss of water, while the dermis and subcutaneous tissue mechanically support the structure of the skin and appendages, including hairs and secretory glands. The integrity of the integumentary system is a key for general health, and many techniques have been developed to measure and control this protective function. In contrast, the effective skin barrier is the major obstacle for transdermal delivery and detection. Changes in the electrical properties of skin, such as impedance and ionic activity, is a practical indicator that reflects the structures and functions of the skin. For example, the impedance that reflects the hydration of the skin is measured for quantitative assessment in skincare, and the current generated across a wound is used for the evaluation and control of wound healing. Furthermore, the electrically charged structure of the skin enables transdermal drug delivery and chemical extraction. This paper provides an overview of the electrical aspects of the skin and summarizes current advances in the development of devices based on these features.

## I. INTRODUCTION

Skin is an intelligent organ, which provides an essential barrier for life on land, regulating internal functions in response to stimulation from the external environment. The electrical properties of this indispensable system have been of interest for decades, especially for practical applications in medical and cosmetic treatments. In particular, the epidermis, the topmost tissue on the surface of the skin, displays its physical structure via its electrical conductivity and physiological ionic distribution in potential difference. Electrical approaches to general biological tissues have been explored for at least several decades, and methods for evaluation and control of the physiological system have been put to practical in both basic research and clinical usage. These methods stand on the fact that the variations in the electrical properties of living tissue represent its structural changes and that emulated ionic signals in the tissue can manipulate the system.^1–8^ These facts have led to the development of electrical methods to assess the integrity of the skin, including wound healing and barrier recovery. Although similar techniques based on these electrical properties have been widely studied in biomedical engineering, there has not yet been a general treatment that covers this field of research.

The unique characteristics and accessibility of skin have inspired the development of various devices. Above all, research on devices called electronic skin (e-skin) has been flourishing. Excellent reviews have been written on a wide variety of such devices and related technologies, including flexible electronics that follow the shape and deformation of the skin, self-healing materials that restore their physical properties, and sensors that detect temperature and pressure.^9–13^ Driven by improvements in material, circuit design, and fabrication technologies, e-skin has revolutionized healthcare and robotics. The operation principles of these devices often depend on the properties of materials and circuits to mimic or replace the functions of the skin. In contrast, the present paper focuses on techniques that work based on the intrinsic electrical properties of the skin, irrespective of the design and concept of the device itself.

**FIG. 1.**
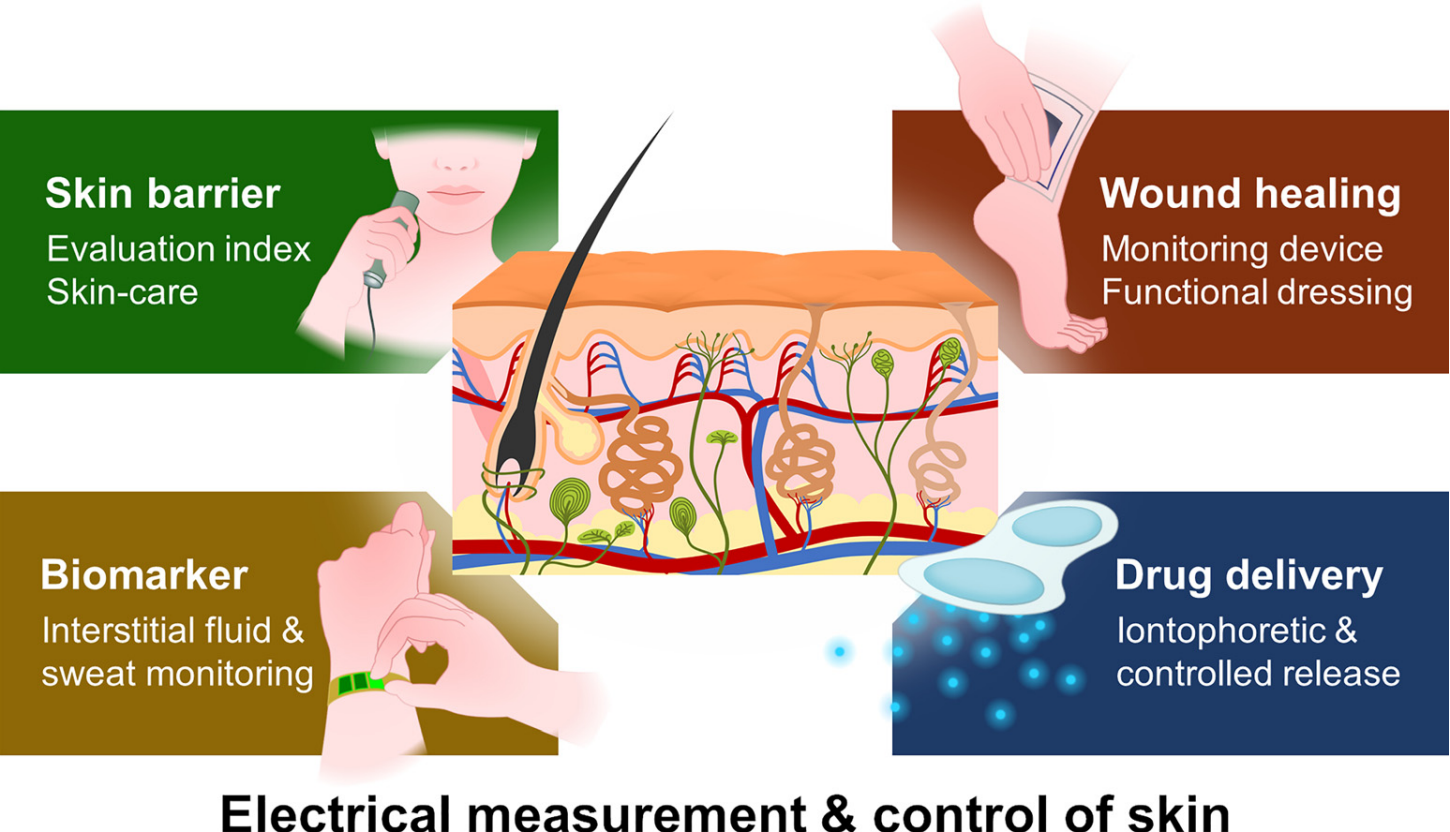
Variety of skin devices covered in this review.

The first part of this paper gives a brief outline of the basic structure and function of the skin, highlighting the major electrical aspects applied in biomedical applications. In the second half, related technologies and devices at the leading edge of research will be presented. The devices presented in this paper are schematically illustrated in Fig. 1.

## II. FUNDAMENTALS IN ELECTRICAL ASPECTS OF THE SKIN

### A. Basic structure and function of the skin

**FIG. 2.**
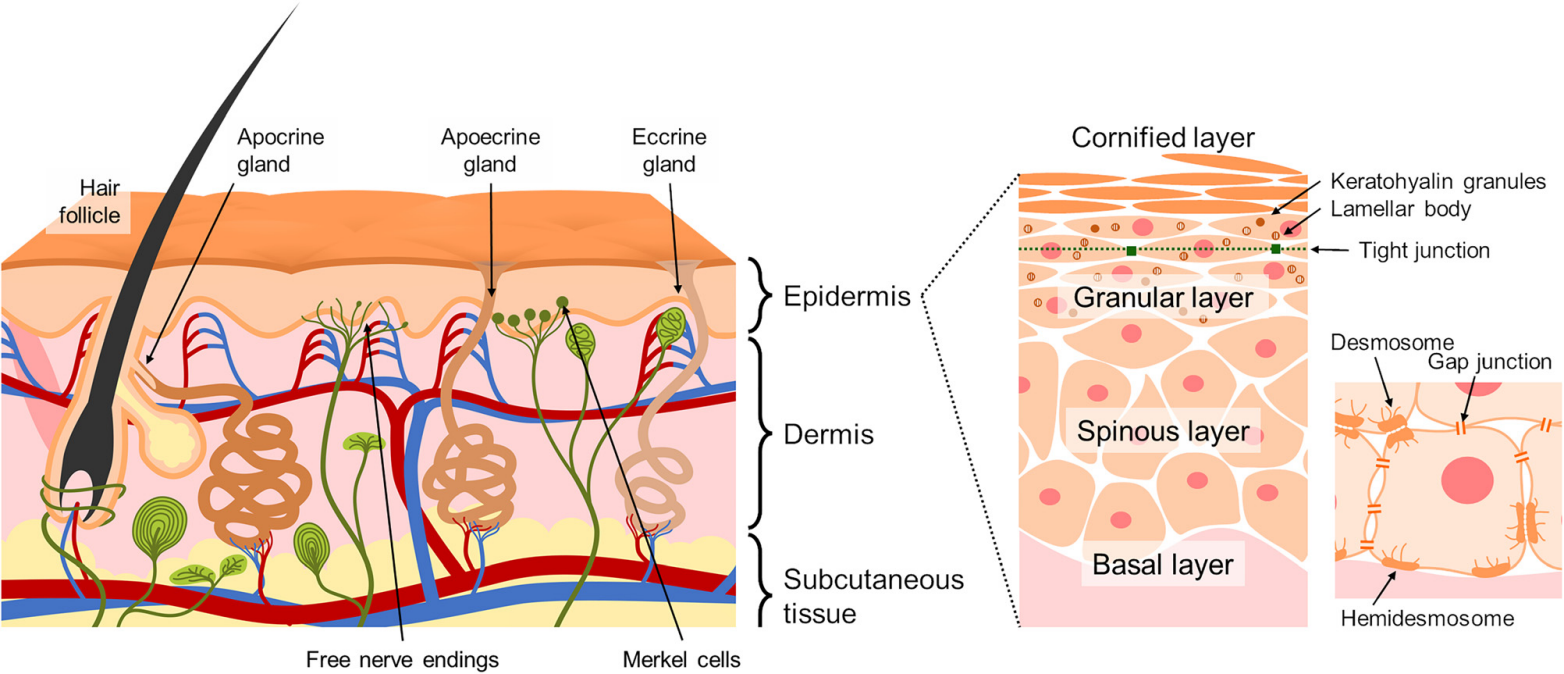
Schematic illustration of the structure of skin.

Human skin is composed of three major layers: epidermis, dermis, and hypodermis (Fig. 2). At the forefront of the living body, they protect inner organs from the outer environment in cooperation. There is a vast and extensive body of research on the structure and function of the skin, and many comprehensive references are available in the literature.^14–16^ Here is a brief description that will help one to understand the electrical properties of the skin.

#### 1. The epidermis

Epidermis is an epithelial tissue mainly composed of keratinocytes, which serves as the skin barrier to cover the entire body to keep external substances out and internal moisture in.^17–19^ Its thickness is about 0.1 mm with variation among sites and individuals^20–22^ and is divided into four layers based on different stages of maturation of tissues. In order from the deepest level, they are called the basal layer (stratum basale), the spinous layer (stratum spinosum), the granular layer (stratum granulosum), and the cornified layer (stratum corneum), and the lower three layers are often referred to as the viable epidermis. Keratinocytes proliferate in the basal layer and then migrate perpendicularly to the outer surface of the tissue undergoing several steps of differentiation. The cells are adhered to each other and to the extracellular matrix below with desmosomes and hemidesmosomes. The gap junction also connects cells together and provides pathways for molecular communication through its junctional channel. In the granular layer, a tight junction is functioning as a diffusion barrier,^23^ and the keratohyalin granules and the lamellar bodies are prepared to maintain the homeostasis of the barrier in the cornified layer above.^24,25^ The final step of the differentiation, or the cornification, induces a drastic change in keratinocytes.^26,27^ The nucleus and cell organelles are degraded, and the strengthened cell bodies and the layers of lipids comprise the dozens of sub-layers that are stacked to provide a protective function against external stresses. The whole process of this terminal differentiation of keratinocytes is estimated to take more than 40 days.^28,29^ The epidermis has a major contribution to the physical electrical properties of skin described below because of its relatively high electrical resistance due to the permeability barrier.

The epidermis also contains a number of cells whose functions include immunity and touch sensation, which interact with keratinocytes.^30–32^ In addition to the physical barrier, keratinocytes are components of the physiological immune response.^33^ A growing number of studies suggest that keratinocytes might also be involved in signal transduction, including the transmission of nociceptive or temperature sensation.^34–38^ Recently, a close association between keratinocytes and free nerve endings was found in the human epidermis.^39^ A detailed review on nociception in the keratinocytes can be found in the literature.^37,38^ As discussed below, the epidermis bears an ionic system analogous to the nervous system, which may expand the electrical applications to regulate these advanced functions of keratinocytes in the future.

#### 2. The dermis

The dermis is the elastic tissue that supports the entire skin mechanically, whose main component is connective tissue formed by collagen and elastin fibers produced by fibroblasts. The structure can be compared to a hydrogel with little cell mass. The dermis serves as a mechanical backing of the skin structure that gives flexibility and elastic strength to the entire tissue,^40,41^ and the increased mechanical mismatch between the epidermis and dermis due to skin aging results in wrinkling.^42,43^ The upper dermis delivers nutrients to the unvascularized epidermal layer via a network of small blood capillaries, which vary their vasodilation state with the condition of the skin, including temperature and inflammation.^44,45^ Numerous nerve endings in the cutaneous sensory system are located in the dermis.^32,46,47^

The dermal fibroblasts play an important role during the wound healing process^48–51^ by the production/degradation of the extracellular matrix components. The interaction with other cell types, such as keratinocytes, at the interface of the different tissues, is being elucidated.

#### 3. The subcutaneous tissue

Underneath the dermis, there is the subcutaneous tissue (also called hypodermis or subcutis) composed of soft connective tissue and adipose tissue supports nerves, blood vessels, lymphatic vessels, and the bases of skin appendages.^52–55^ The largest component of this layer is aggregations of adipose cells divided into small lobules by septa, the connective tissue extending throughout the tissue along with the vessels and the nerves to fix the layer with the dermis. The distribution and components of the adipose tissue vary depending on the location and between individuals. The matrix physically absorbs external forces and heat while physiologically contributing to metabolic function.

#### 4. The skin appendages

Hair follicles and sweat glands are the commonly distributed appendages throughout the skin.

The bottom of the hair follicle is located in the dermis or subcutaneous tissue, and the hair shafts are surrounded by layers of sheath and sebum secreted by sebaceous glands. The inner surface of the opening (infundibulum) was found to be covered by the stratum corneum, which is similar to that in the epidermis, and a continuous tight junction was formed from the opening to near the bulb.^56^ This tight junction barrier is suggested to be not as tight as the epidermal barrier, indicating that the hair follicles are a potential shunt pathway for drug delivery.^57^

There are three types of sweat glands: eccrine, apocrine, and apoecrine glands. The eccrine and apoecrine glands, which mainly secrete aqueous solution, have ducts that open directly to the surface of the skin, while the ducts of the apocrine glands, which secrete a viscous solution containing proteins and lipids, are connected to the hair follicle, and all types have secretion coils located in the deep dermis or the subcutaneous tissue.^58^ Sweat glands also form tight junctions, which are considered to be a barrier to separate the inner tissue from the outer environment.^59,60^ In particular, the eccrine glands are the most common type distributed throughout the body, numbering of up to four million,^61,62^ and are also expected to provide the shunt pathways to access into the skin.

### B. Electrical features of the skin

The electrical properties of the skin are of primary importance in engineering applications. For instance, the physical properties such as the resistance and capacitance reflect the structure of the skin. Since many bioelectrical measurement methods (e.g., electrocardiogram, electroencephalogram, electromyography), track the electrical phenomena inside the body via the skin surface electrodes, the physical properties of the skin inevitably affect the obtained signal. Meanwhile, these measurable properties can serve as indicators for quantitative electrical evaluation of structural changes that occur under the influence of the external environment or diseases.

Moreover, the electrical features of the skin involve physiological properties and physical properties. As with other electrically active tissues (i.e., neurons and muscles), ionic signaling is essential in maintaining homeostasis in the skin. Advances in the study of the physiological properties are further expanding the range of applications of the technologies from evaluation to control.

Here, we highlight the electrical properties and related activities of the skin. This section begins by presenting the physical properties and their application to bioengineering. Subjects concerning the ionic distribution and potential difference generated across the epidermis, including electrotherapy inspired by the endogenous electrical field, are described later. Furthermore, iontophoresis is also reviewed as the method utilizes the electrically charged structure of the skin. An outline of electrical stimulation to the skin appendages is given at the end of the section.

#### 1. Physical properties

**FIG. 3.**
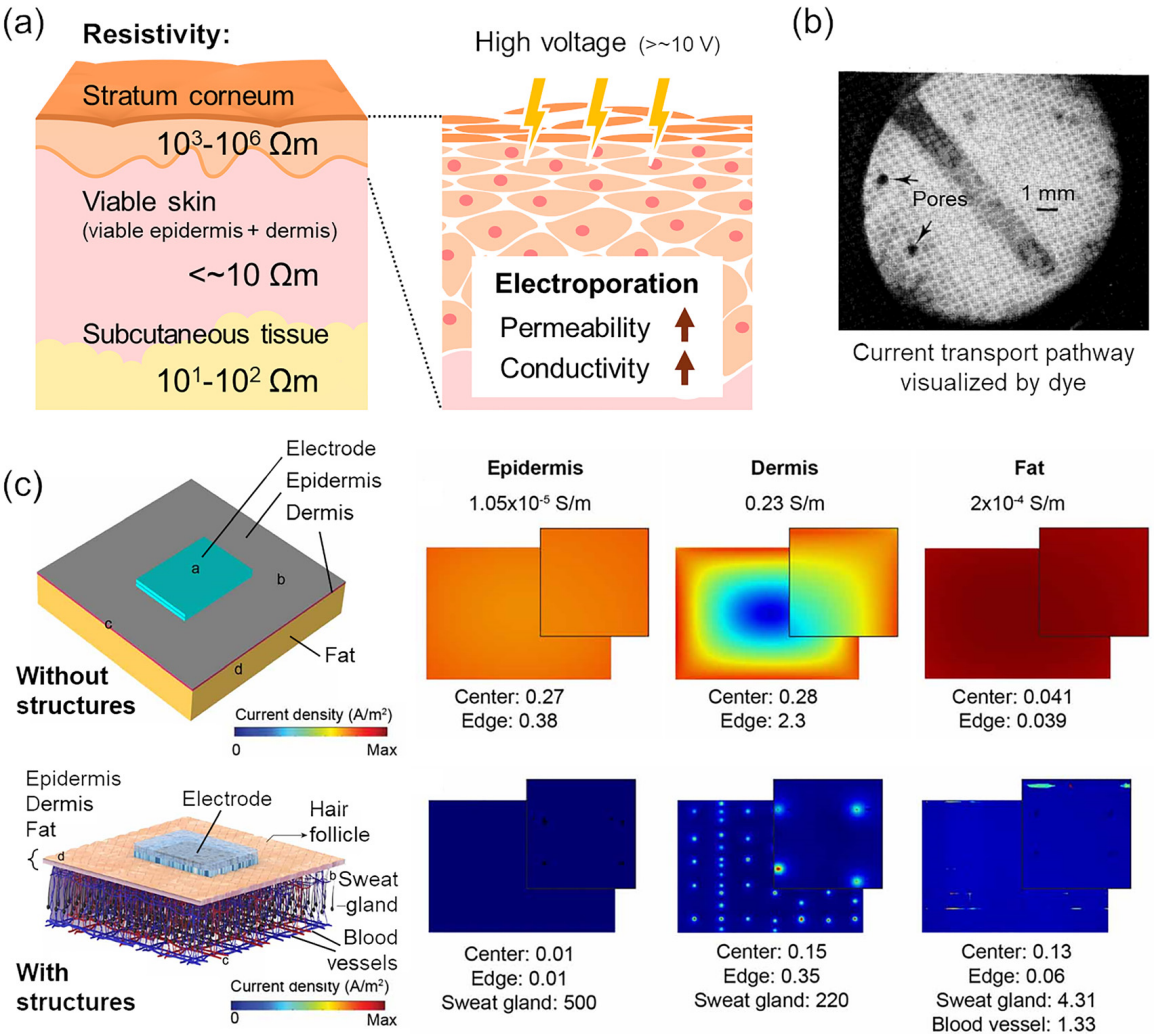
Passive electrical properties of the skin. (a) The resistivity of each layer of skin. At relatively high voltages, electroporation increases the permeability and conductivity of the skin. The literature values are from Refs. 63 and 66–70. (b) Localization of staining fluids carried by iontophoretic (see below) current with an applied current density of ca. 0.16 mA/cm^2^. The pore-like distribution suggests shunt pathways for the electrical current. Adapted with permission from Burnette and Ongpipattanakul, J. Pharm. Sci. **77**, 2 (1988).^87^ Copyright 1988 Elsevier. (c) Simulated current distribution in the skin model with and without hair follicle, sweat gland, and blood vessel structures. The dermal model with these structures resulted in the patterned current density peaked around the appendages. Adapted with permission from Khadka and Bikson, Phys. Med. Biol. **65**, 22 (2020).^92^ Copyright 2020 Institute of Physics and Engineering in Medicine.

In the skin tissue, the applied current is transported by the charged water-soluble molecules inside, and therefore, the stratum corneum primarily affects the overall impedance due to its hydrophobic nature as the permeability barrier. The underlying tissue with higher moisture content accounts for a smaller impedance. Equivalent circuits of biological tissues are commonly represented as a parallel circuit of resistance and capacitance (sometimes replaced by constant phase element, CPE, that can approximate complex system into the simple circuit) associated with ionic components and mobility, and skin models consisting of different skin layers are often modeled as a combination of them.^63–66^ In every method for measurement and treatment with the transdermal current flow, the impedance characteristics are crucial for appropriate system design. The magnitude of the direct current (DC) or low–frequency resistivity of the stratum corneum is estimated to be 10^3^–10^6^ Ω m,^63,67–69^ while that of underlying viable skin (including the viable epidermis and the dermis) and subcutaneous fatty tissue might be a few Ω m (Refs. 63, 66, and 68) and 10^1^–10^2^ Ω m,^68–70^ respectively [Fig. 3(a)]. It is worth noting that the values vary with the degree of hydration and ionic concentration and between individual subjects.^64,67,69,71^ Similar to other biological tissues, the skin has a dielectric property, and its resistivity is high at low frequency and decreases with dispersion due to its capacitive characteristics at high frequency. The conductance or the capacitance at high frequency (typically on the order of MHz) is measured with a dry-contact planar electrode^72,73^ or needle-like electrodes^74,75^ to evaluate the hydration state of the skin surface. In general, the conductance and the capacitance increase in hydrated skin, while the impedance changes in the opposite direction. Compared to youthful normal skin, the values are larger in erosive lesions and scars, while they are smaller in scaly lesions and aged skin.^73,76^ Interestingly, transepidermal water loss (TEWL), a standard index of skin barrier function that represents the amount of water passes through the stratum corneum, increases in pathologic dry skin and decreases in aged dry skin.^76^ Electrical measurements can provide a more accurate assessment of the skin function when used in combination with other techniques. Moreover, the application of relatively high voltage (>ca. 10 V on the skin) increases the permeability of the cells by electroporation^77^ and induces the transient or irreversible change in molecular dynamics due to thermal perturbation and electroosmosis,^78–80^ hence, increases the conductivity of the skin [Fig. 3(a)].^79–82^ Those physical effects are utilized to enhance the penetration of a drug into the skin, as reviewed in Refs. 83–85.

Although the skin can be regarded as a homogeneous matrix or a stack of layers of different matrix components for simplicity, several experiments^86–90^ and models^91,92^ show that the in-plane distribution of the current density is considerably uneven on account of the dispersed pores with high conductivity [Figs. 3(b) and 3(c)], including the appendages, especially in the case of low frequencies and low voltages. In contrast to the cornified layer which restricts the movement of water, hair follicles and sweat glands with relatively weak permeability barriers and aqueous secretions are more likely to be a preferred path for electrical currents. This gives an important insight for designing efficient electrical systems minimizing adverse effects such as burns, irritation, and erythema.

#### 2. Ionic distribution

**FIG. 4.**
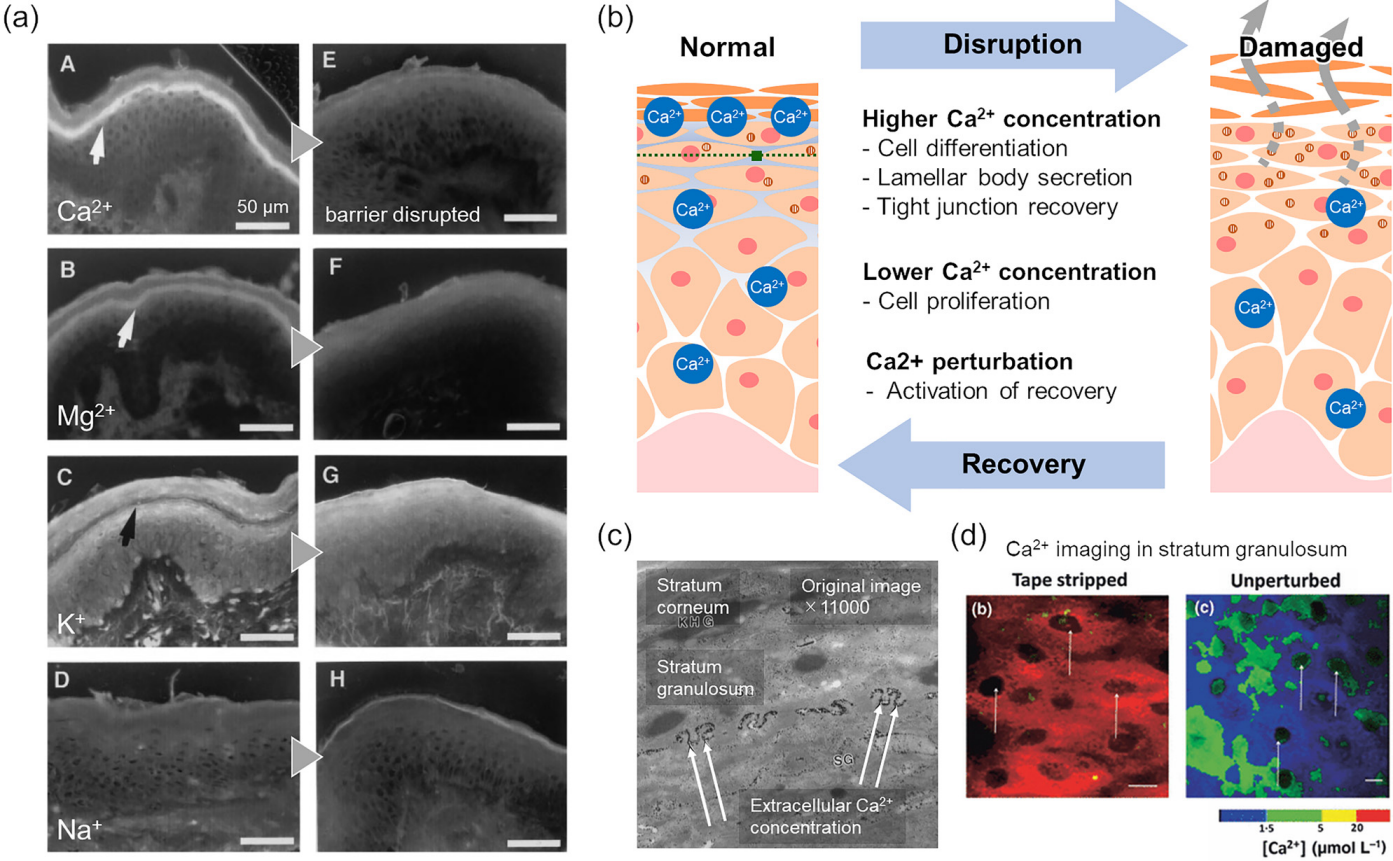
Ion distribution in the viable epidermis. (a) Distribution of various ion species (Ca^2+^, Mg^2+^, K^+^, Na^+^) in human epidermis before and after barrier disruption visualized by fluorescent imaging. Arrows indicate the localized area. Adapted with permission from Denda *et al.*, Biochem. Biophys. Res. Commun. **272**, 1 (2000).^95^ Copyright 2000 Elsevier. (b) Schematic illustration of calcium modulation of epidermal function. Calcium localization in the normal epidermis promotes proliferation in the lower epidermis and differentiation in the upper epidermis. Perturbation of the distribution by damage triggers recovery. (c) Visualization of calcium aggregation in stratum granulosum of the murine epidermis. Arrows indicate calcium precipitation collected in the intercellular space. Adapted with permission from Menon *et al.*, Cell Tissue Res. **270**, 3 (1992).^102^ Copyright 1992 Springer-Verlag. (d) Calcium imaging of keratinocytes in stratum granulosum of the murine epidermis. Signals, which indicate high Ca^2+^ concentration originated from intracellular compartments, were lost by skin barrier disruption caused by tape stripping. Adapted with permission from Celli *et al.*, Br. J. Dermatol. **164**, 1 (2011).^137^ Copyright 2010 John Wiley and Sons.

Comparable to the electrically active tissues, including neurons and muscles, the inorganic ions play specific roles in the skin. Intact epidermal tissue maintains a nonuniform ion distribution, and the determination or the visualization of the individual species has received considerable attention for decades. Since the epidermis is composed of a dense aggregate of keratinocytes, the more cellular ion transport and the less diffusion than in the underlying tissues are expected. Multiple studies showed divalent ions such as calcium and magnesium have prominent peaks above the stratum granulosum at the border of the viable epidermis and the stratum corneum,^93–95^ while sodium and chloride were distributed in a moderate gradient over the viable epidermal layer,^94–97^ and potassium was reduced from the stratum granulosum to the lower layers [Fig. 4(a)].^94–97^ The ionic profile of the outer stratum corneum is reported to be susceptible to the penetration of external substances;^98–101^ hence, the ionic distribution below the stratum corneum will be discussed in this paper. The distribution of ions in the viable epidermis is changed according to acute and chronic barrier perturbation,^95,102,103^ diseases associated with abnormalities in barrier and differentiation,^104–108^ and wounds.^109–111^

Among them, calcium has been investigated as an important signal, as its distribution is consistent with different demands of each stratum at specific stages of differentiation. Lower concentrations in the lower layers promote proliferation, whereas higher concentrations in the upper layers promote differentiation and lamellar body secretion to form the functional skin barrier.^102,112–116^ Loss of calcium localization in the stratum granulosum triggers barrier recovery processes, including lamellar body secretion to fill the intercellular spaces of the stratum corneum with lipids.^102,115,117^ In addition, calcium is required for cell migration and desmosome formation in the wound-healing response of epidermal wounds.^118–121^ Along with the cell migration essential for wound closure and re-epithelialization, desmosomal adhesion is considered to be controlled in a calcium-dependent manner to allow cell migration in the early stage in wound healing and to provide mechanical support for the regenerated epithelium later. The change in the ionic profile due to acute barrier perturbation and wounds is normally restored as the structure of the skin recovers.^102,109,110^ These observations suggest that the changes in ionic dynamics serve as a signal for skin tissue to detect itself as damaged [Fig. 4(b)].

Conversely, the epidermal response can be triggered by manipulating the ion profile. The increased influx of calcium ions into keratinocytes by the topical application of ionophores perturbed lamellar body secretion, hence hindered barrier recovery.^122^ An altered calcium gradient as a result of water flux into the skin generated by the application of electrical current or ultrasound (i.e., iontophoresis/sonophoresis) induced epidermal responses including lamellar body secretion, as seen in the actual barrier breakdown.^114,123,124^ Immersion in electrolytic solutions has also been reported to inhibit or accelerate the barrier recovery process, depending on the ionic species and composition ratio, suggesting that the addition of calcium to the tissue that has otherwise lost calcium localization disrupts the endogenous barrier recovery process, while the combination of calcium and magnesium may serve as a signal for epidermal homeostasis.^125–127^ Methods to prevent contractures in wound or skin graft by reducing divalent ions to regulate the keratinocyte proliferation and differentiation that affect the properties of regenerated tissue have also been reported.^128,129^

Early studies have given emphasis to extracellular calcium aggregation; ion-capture cytochemistry has suggested that the calcium localization was in the intercellular gap in the stratum granulosum [Fig. 4(c)].^93,102,130^ In agreement with these observations, the tight junction barrier in the stratum granulosum forms a barrier for ions^131^ that is essential for calcium localization associated with normal growth and differentiation in the epidermis.^132^ The mechanisms underlying the disruption and recovery of the calcium localization that attended barrier perturbation are regarded as partly passive processes. The experimentally disrupted calcium gradient in the barrier-disrupted epidermis was restored by restricting the water flux with a vapor-permeable membrane, implying the movement of extracellular fluid.^133^ However, neither calcium localization nor the barrier was restored when the skin was completely occluded with a vapor-impermeable membrane,^103,134,135^ suggesting that the recovery was not completed solely by stimulation by calcium perturbation, but required another signal. In recent years, an evaluation method focusing on free calcium that is resistant to artifacts has become available,^121^ and the major contribution of intracellular calcium stored in the organelles to the calcium dynamics in the tissue was reported [Fig. 4(d)].^121,136–138^ In another study, the corneal epithelium, which has a multilayered epithelial structure similar to that of the epidermis, was hired as a wound model, and the results suggested that not only does ion leakage occur in wounds due to structural breakdown, but there is also a physiological response of active ion transport.^139^ These examples indicate possible applications of the electrical signaling of the skin.

#### 3. Transepidermal potential difference (TEP)

Interestingly, a potential difference called transepidermal potential (TEP) is generated in the thickness direction of the epidermis,^140,141^ probably due to the gradient of charged species across the tissue. TEP is negative on the surface of the skin, and its magnitude is up to tens of millivolts with regional variations. Initial studies primarily focused on the relationship between the various electrical characteristics of the skin, including the electrical potential and psychological response^142^ attributed to the activity of the sweat glands as summarized in the references cited.^143,144^ Later, the independently generated potential difference across the epidermis was found^140^ and the involvement of the physiological system and the ion dynamics in epidermal keratinocytes.^145^

**FIG. 5.**
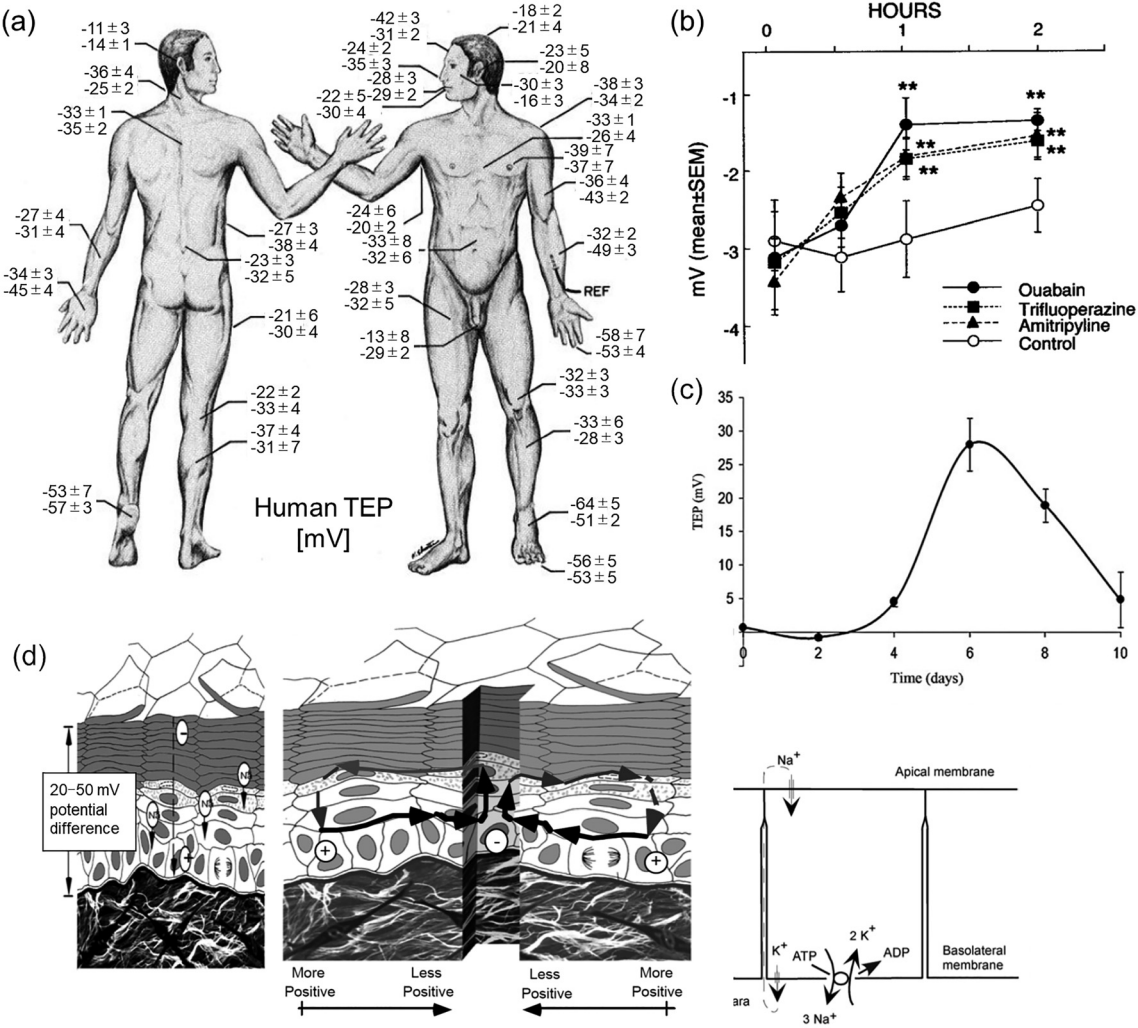
Transepidermal potential difference (TEP). (a) Measured TEP in human skin against the reference positioned in subdermal space in the lower arm. Adapted with permission from Barker *et al.*, Am. J. Physiol. Integr. Comp. Physiol. **242**, 3 (1982).^140^ Copyright 1982 American Physiological Society. (b) The change in TEP after the application of various ion channel inhibitors to the extracted skin of hairless mice. 50 *μ*M of Ouabain (used as the inhibitor of Na^+^/K^+^ ATPase), trifluoperazine, and amitriptyline (used as the indirect inhibitor of Ca^2+^, Mg^2+^ ATPase) decreased TEP (^**^P < 0.01). Reproduced with permission from Denda *et al.*, Biochem. Biophys. Res. Commun. **284**, 1 (2001).^145^ Copyright 2001 Elsevier. (c) The change in TEP during wound reepithelialization on the *in vivo* porcine skin. 3 Landrace–Yorkshire female pigs were used, and six wounds (6 mm in diameter and 2.5 mm in thickness) were created. The attenuated TEP at day 0 after the creation of the wound rapidly increased and decreased according to time. Reproduced with permission from Dubé *et al.*, Tissue Eng., Part A **16**, 10 (2010).^146^ Copyright 2010 Mary Ann Liebert, Inc. (d) Schematic diagram of TEP and wound current generation. The voltage inside the intact epidermis is considered to derive from net cation influx (sum of Na^+^ influx and K^+^ efflux). The short-circuit in the wound results in the lateral current. Adapted with permission from Nuccitelli, Curr. Top. Dev. Biol. **58**, 1 (2003).^149^ Copyright 2003 Elsevier Science Ireland Ltd.

On intact epidermis, measured values of TEP have been reported as 10–60 mV, depending on the anatomical site [Fig. 5(a)].^140,141^ Almost no potential difference was found below the dermis, where the ionic gradients are barely maintained due to relatively sparse tissue.^140^ Since TEP has been reported to be attenuated by metabolic inhibitors, and calcium, potassium, and sodium channel blockers [Fig. 5(b)],^145^ active transport of ions in the viable epidermis is presumed to be involved in the potential generation. Other factors reported to reduce TEP are skin injury [Fig. 5(c)],^140,146^ permeability barrier perturbation,^147,148^ and aging,^148^ all of which cause functional alterations in the epidermal barrier. The decrease was transient in the wound or barrier disruption, and the values were returned to their original level along with the recovery process, which may take hours or days. Those changes may consist of both passive diffusion and active transport occurs in the tissue, but the detailed mechanism is still under investigation.

The role of TEP in promoting wound healing has been a topic of interest for decades^8,140,141,149–152^ and is one of the most well-studied electrical features of the skin. In the skin injury cases where the tissue is structurally destroyed, TEP is short-circuited in the exudate (wound fluid) to generate an endogenous current of several *μ*A/cm^2^ from the margin to the center of the wound bed [Fig. 5(d)].^140^ Several types of cells including epidermal keratinocytes, dermal fibroblasts, and other immune cells involved in wound healing show galvanotaxis and migrate toward the cathode in an applied electrical field^152,153^ in a direction to close the wound. *In vitro* experiments on epithelial cells have shown that electrical stimulation predominantly directed their migration, overriding other cues possibly, including chemical signals and mechanical guidance produced by the wound.^154,155^ The potential difference across the wound recovered with a certain time course till the achievement of wound closure and tissue maturation.^146,156^ As mentioned above, efflux of calcium and potassium and influx of sodium and chloride were observed in the *ex vivo* model of corneal epithelium, and it has been surmised that both ion leakage and active ion transport are involved in the potential change.^139^ In addition, other processes in wound healing, including cell proliferation, blood perfusion increase, and tissue matrix production, have been revealed to be electrically promoted.^8,151,152^

Enhancement of these effects by external stimuli has also been tested for decades. Although the nature of the endogenous electric field suggests that DC stimulation in a constant voltage application should well imitate the *in vivo* environment, pulsed current is reported to promote keratinocyte migration similar to that seen with constant current stimulation.^157^ Pulsed current enables a higher voltage, which may cause tissue damage in DC due to electrolysis or heating,^158^ yet more comparable studies focused on this issue are needed to provide firm conclusions. The modality of stimulation should be determined concurrently with the development of treatments and devices.

#### 4. Transdermal iontophoresis

**FIG. 6.**
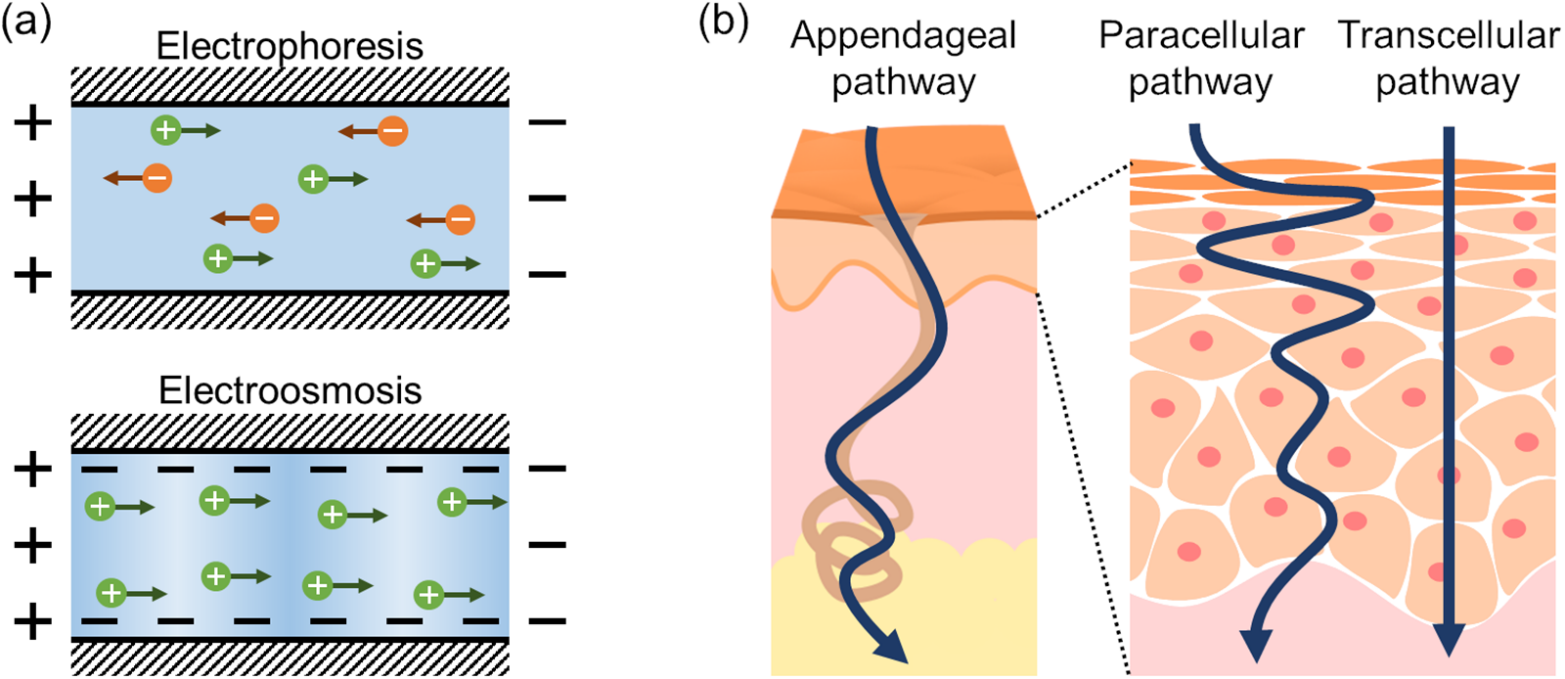
Transdermal iontophoresis. (a) Schematic illustration of the two major mechanisms, electrophoresis and electro-osmosis. In electrophoresis, the charged species in the solution move along the applied electric field according to their charge. In electro-osmosis, the counterions inside the charged microstructure drag the solvent as they move along the applied electrical field and generate the net solution flow. (b) Schematic illustration of the current pathway across the skin. The appendages serve as hydrophilic shunt pathways to bypass the lipophilic epidermis, paracellular, or transcellular pathways.

Iontophoresis is a method to control or enhance the molecular flux across the skin with the electrical current application, which generally employs a lower current (typically < 0.5 mA/cm^2^) than used in electroporation. In contrast to the stratum corneum prevents the passage of external and internal substances, the underlying tissues allow them to diffuse and transport into the blood flow, thus this technique makes the skin into the more practical pathway for molecular transport to the whole system. It has long been studied chiefly as a method of transdermal drug delivery, offering the advantages of a programmable delivery profile while expanding the range of species that can be dosed noninvasively.^159–161^ The major mechanisms are electrophoresis and electro-osmosis [Fig. 6(a)]. Electrophoresis is the migration of charged species along the electric field toward the electrode of the opposite charge. The mobility of permeants in the matrix depends on their physicochemical characteristics such as size, charge, and polarity. In transdermal iontophoresis, the matrix is skin tissue with a net negative charge at physiological pH.^162^ The counterion inside the charged microstructure moving along the electric field drag solvent, hence net solution flow i.e., electroosmotic flow is induced in the direction of counterion migration. In skin tissue, the flow is generated from the anode to the cathode in the direction of the cation movement, facilitating the delivery of cationic and neutral solutes. The contribution of electrophoresis and electro-osmosis to the flux depends on the physicochemical and electrical characteristics of both matrix and solution. It has been reported that electroosmotic flux can be dominant even with anions for delivery from the anode^163^ and can be modulated by lowering the pH^164^ or introducing molecules^165,166^ to change the permselectivity of skin tissue. This flow is also used in the opposite direction to extract interstitial fluids from the skin tissue for analysis of biomarkers.^167,168^ The interstitial fluid carries biomarkers derived from the vascular network in the dermis, but it is kept below the epidermal barrier; thus, the technique to extract it from intact skin would be a desirable alternative to blood sampling. For example, the correlation between the glucose concentration in the interstitial fluid and the blood^169–171^ has encouraged glucose monitoring by “reverse iontophoresis.”

The benefit of this method is the active control of drug delivery. The transdermal pathway is advantageous over oral administration and injection because it avoids degradation in the digestive system and pain. Various methods have been explored to break through the skin barrier for a wider scope of drugs.^172,173^ Advances have been made in various methods, including microneedle,^174,175^ sonophoresis (ultrasound irradiation to collapse the microstructure of the stratum corneum by cavitation),^176,177^ and thermal cauterization (local heating of the skin surface by laser, high-frequency electric field, or other heat sources to create pores)^178,179^ in addition to electroporation. Since these methods induce physical alterations in the stratum corneum, they are applicable to a relatively wide variety of molecules. These techniques are relatively broad in scope because they induce physical alterations in the stratum corneum, but drug transport is passively driven by diffusion, and the amount and timing of delivery cannot be controlled. In contrast, iontophoresis allows the programmed delivery, but its mechanism limits the characteristics of drugs that can be effectively delivered; thus, methods that combine multiple techniques are being explored.^180,181^

Iontophoretic transport is assumed to occur mainly through the appendageal pathway, which can be inferred from the fact that the distribution of the current density within the skin is not uniform, resulting from the uneven resistivity described above.^86–92^ Delivered permeants have been reported to be localized in the hair follicle or sweat duct, especially for relatively hydrophilic species, while the contribution of paracellular or transcellular pathways in the stratum corneum and viable epidermis as lipophilic diffusion pathway is limited [Fig. 6(b)].^182–184^ In accordance with these observations, pores in isolated human stratum corneum^184^ nor reptilian skin without any appendage^183^ generated no iontophoretic flow. On the contrary, excessive perforation by electroporation is reported to diminish the effect of iontophoresis,^185^ and the electroosmotic flow of both hydrophilic and lipophilic permeant was considerably reduced in a skin sample where the stratum corneum has been removed by tape stripping,^164,186^ suggesting the involvement of the structure of normal stratum corneum in iontophoretic transport. It should be noted that invasive pretreatment can lead to perturbation of the function in the living skin. Since iontophoretic stimulation was recently suggested to induce a physiological response to attenuate gap junction in the epidermis and increase endocytosis,^187–189^ further studies *in vivo* or in a comparable model will be essential for better understanding of both physical and physiological processes.

In this regard, the permeability of the skin tissue is known to be increased during iontophoresis.^190^ It is partially due to the damage caused by a topical high current density^89^ and hydration by occlusion under the electrode,^123^ serving as another transport pathway. The change in electrical impedance mainly reflects the change in ion distribution in the stratum corneum and viable epidermis.^64,191,192^ As mentioned above, iontophoresis considerably changed the calcium distribution in the epidermis to activate the barrier recovery process.^123,124^ Transdermal voltage application using similar settings as the iontophoresis is reported to accelerate barrier recovery and modulate calcium ion dynamics,^127,193^ which has led to the extension of the application of charged particles^194^ or metal^195^ onto the skin surface.

#### 5. Electrical stimulation to the skin appendages

As an electrical treatment for sweat glands, iontophoresis of tap water has been used for decades as an effective therapeutic option for palmoplantar hyperhidrosis.^196–199^ Commercially available devices approved by the FDA, DERMADRY^^®^^ (Dermadry Laboratories Inc.), Drionic^®^ (GENERAL MEDICAL CO.), HIDREX^®^ (HIDREX GmbH), and The Fischer (Saalmann medical GmbH & Co. KG), have pads or chamber to hold tap water, through which the affected area is stimulated. Typically, the affected area is placed in contact with the tap water for 20–30 min while the electrical current of up to ca. 20 mA is applied. The treatment should be repeated once to three times a week to maintain the anhidrosis (absence of perspiration). This noninvasive, nondrug method is promising to minimize adverse effects.

The mechanism of action of iontophoresis on sweating is still unknown. Although sweat pore blockage by a hyperkeratotic plug was observed in areas other than the palmoplantar region,^200–203^ this structural change was not reproduced on the palm skin,^204^ nor the morphological change related to the nervous system that controls sweating.^205^ The pH change under the stimulation electrode is suggested to inhibit the sweat gland activity.^206^ Also, the concentration of sodium ions in sweat was decreased after alternating current (AC) iontophoresis, suggesting the modulation of secretion and reabsorption process in sweat glands.^207^

Hair follicles are also suggested to be sensitive to electrical stimulation. The application of pulsed electrical field is reported to be effective on male-pattern hair loss (one of the common hair disorders that affects both males and females)^208–211^ and hair loss in cancer chemotherapy^212^ in clinical studies. Although this method seems to be a promising treatment with minimal adverse effects,^213^ little is reported about the detailed parameter settings for stimulation or their optimization, and thus very little research on device development. Relevant *in vitro* and *in vivo* studies have shown that some cations had inhibitory effects on the enzyme involved in the regulation of hair growth^214^ and AC stimulation induced proliferation of follicular cells and related gene expression.^215^ Further studies are needed to elucidate underlying mechanisms and to characterize the effects of various stimulation modalities.

## III. SKIN DEVICES BASED ON THE ELECTRICAL FEATURES OF THE SKIN

Although there are many bioelectrical methods that make contact with internal organs through the skin, this paper focuses on devices that directly affect the skin itself based on the electrical features described above. Specifically, the existing devices and the latest research, especially on wearable devices are reviewed, which are intended for evaluation and control of skin surface properties and wound healing, as well as transdermal drug delivery and biomarker extraction. Table I lists the objectives, the electrical features, and the type of the devices presented in this section. The physical properties of the skin (conductivity, capacity) measured by skin surface electrodes are used as the index of skin hydration level. TEP across the epidermis or its distribution measured on the surface reflects the integrity of the skin. Inspired by the recovery-promoting effect of endogenous TEP, wound dressing devices featuring electrical stimulation have also been developed. In addition, the application of voltage to the skin induces directional molecular transport through charged microstructures, allowing drug delivery into the skin or molecular extraction out of the skin with wearable devices.

**TABLE I.** The objectives, electrical features, and types of skin devices.

Objective	Electrical feature	Type of device	References
Evaluation of skin surface hydration	- Conductivity	- Desktop apparatus of portable probe	76, 219, 220
	- Capacity	- Wearable patch	221–230
Evaluation of skin barrier	- TEP	- Desktop apparatus of portable probe	231–233
		- Wearable patch	234
Evaluation of wound	- TEP	- Portable probe	156, 235
Promotion of wound healing	- TEP	- Patch wired to desktop apparatus	236
		- Wireless patch with built-in power source	237–242
Transdermal drug delivery	- Electro-osmosis via charged microstructure	- Patch wired to desktop apparatus	159, 161, 275–288
		- Wireless patch with built-in power source	
Transdermal molecular extraction	- Reverse electro-osmosis via charged microstructure	- Patch wired to desktop apparatus	289–302
		- Wireless patch with built-in power source	

### A. Evaluation of the skin surface characteristics

**FIG. 7.**
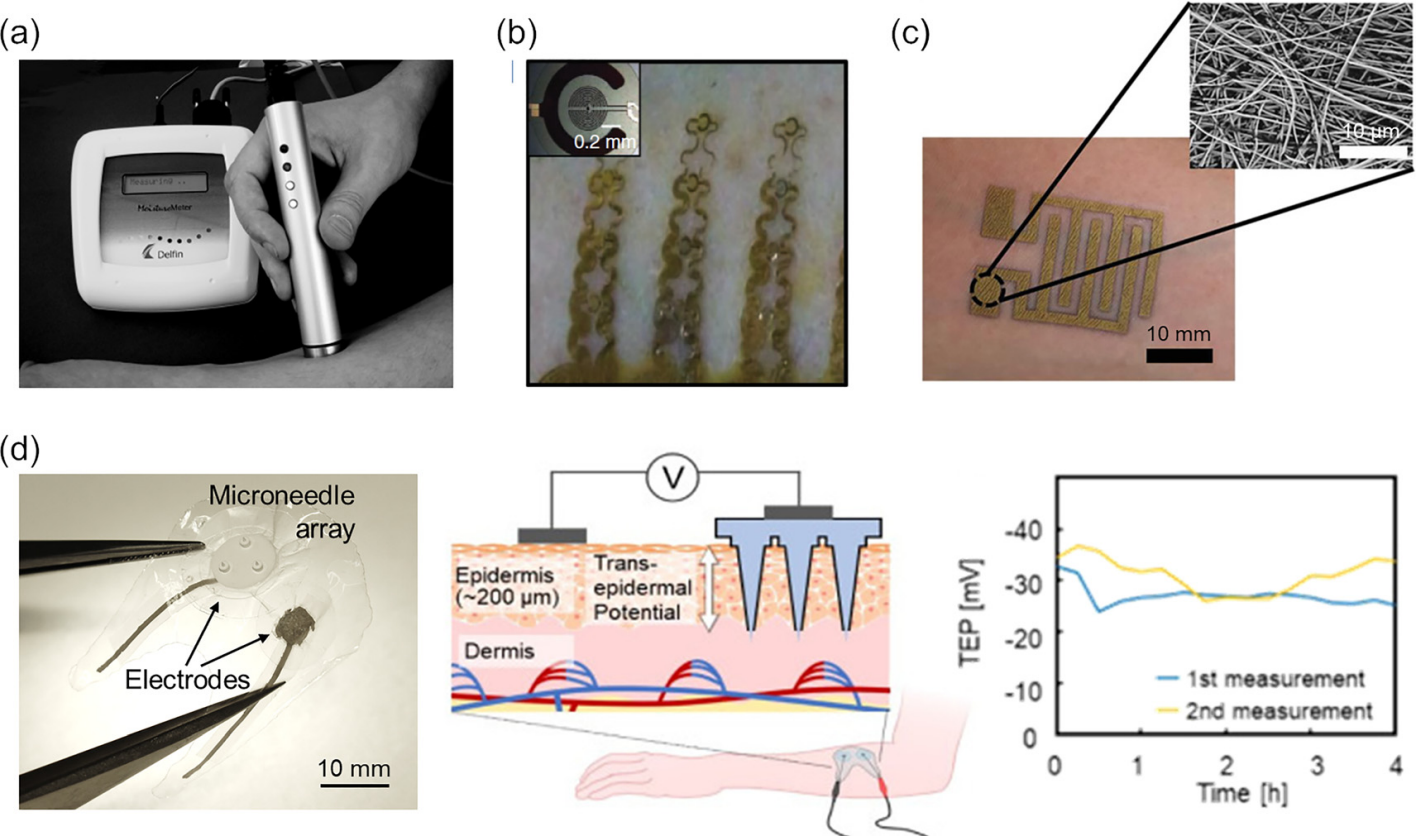
Devices for electrical evaluation of the skin barrier. (a) MoistureMeterSC, which measures the dielectric constant of the skin surface using a coaxial probe. Reproduced with permission from Alanen *et al.*, Skin Res. Technol. **10**, 1 (2004).^220^ Copyright 2004 John Wiley and Sons A/S. (b) Wearable concentric electrodes for skin impedance evaluation. The concentric electrodes are connected by flexible serpentine wires. Thermal and electrical sensing elements are integrated to perform the multimodal measurement. Reproduced with permission from Krishnan *et al.*, Microsyst. Nanoeng. **3**, 1 (2017);^229^ Copyright 2017 Author(s), licensed under a Creative Commons Attribution (CC BY 4.0) License. (c) Wearable comb-shaped electrodes for skin impedance evaluation. The electrodes have a mesh structure deposited on an electrospun sheet. Reproduced with permission from Matsukawa *et al.*, Adv. Healthcare Mater. **9**, 22 (2020).^230^ Copyright 2020 John Wiley and Sons. (d) Minimally invasive TEP measuring patch. One of the electrodes is electrically connected to the subepidermal tissue via a microneedle array. The wearable device enabled TEP monitoring for hours. Adapted with permission from Abe *et al.*, Biomed. Eng. Adv. **1**, 100004 (2021).^234^ Copyright 2021 Author(s), licensed under a Creative Commons Attribution (CC BY-NC-ND 4.0).

As mentioned earlier, hydration of the skin surface can be evaluated based on its conductivity or capacity. Because skin barrier–related disorders are often accompanied by dehydration that causes cracking or scaling on the skin surface,^216–218^ this evaluation technique has long been the focus of attention as a quantitative assessment. Various devices are commercially available to evaluate skin hydration; Skicon^®^ (YAYOI Co., Ltd.) and MoistureMeterSC (Delfin Technologies Ltd.) [Fig. 7(a)] that evaluate the conductance and permittivity, respectively, by a coaxial probe, and Corneometer^®^ (Courage+Khazaka electronic GmbH) that measure the capacitance by a comb electrode are such examples.^76,219,220^ Several wearable devices to monitor skin hydration as electrical conductance or impedance have been developed in which fine electrodes ensure firm electrical contact and air permeability at the same time.^221–228^ For instance, Krishnan *et al.* integrated concentric electrodes and snake-shaped wires into a thin elastomeric substrate [Fig. 7(b)],^229^ and Someya *et al.* used mesh electrodes deposited on an electrospun sheet^230^ to evaluate *in vivo* skin hydration as changes in impedance [Fig. 7(c)].

Although the TEP is also suggested to be correlated with the barrier function,^147,148^ its application to barrier evaluation is still in the early stages of research, and practical devices are not yet available in the market. One of the difficulties is the invasiveness accompanying the electrical connection into the skin, since the TEP is inherently generated along the thickness direction of the skin. Recently, a minimally invasive device for the TEP measurement has been developed and succeeded in the detection of the response to physical stimulation and the evaluation of therapeutic effects on the skin.^231–233^ The device used a thin injection needle filled with an electrolyte solution for ionic conduction to the subepidermal region, enabling local measurement of potential differences across the epidermis. In one of these studies, which evaluated the promotive effect of light stimulation in barrier recovery, the experimental barrier breakdown and subsequent recovery were successfully observed as the change in TEP. Moreover, the authors have developed a wearable device based on printable electrodes equipped with microneedle and successfully monitored TEP for several hours [Fig. 7(d)].^234^ As mentioned above, electrical stimulation may promote barrier recovery,^127,193^ suggesting the possibility of a multifunctional device that performs both evaluation and control of the epidermal barrier. Further elucidation of the mechanism and validation of the effects of the device will be required to develop a device that can be applied to treat chronic skin abnormalities associated with reduced barrier function.

### B. Monitoring and promoting wound healing

Based on a vast research history, there are numerous examples of devices that approach the wound healing process.

**FIG. 8.**
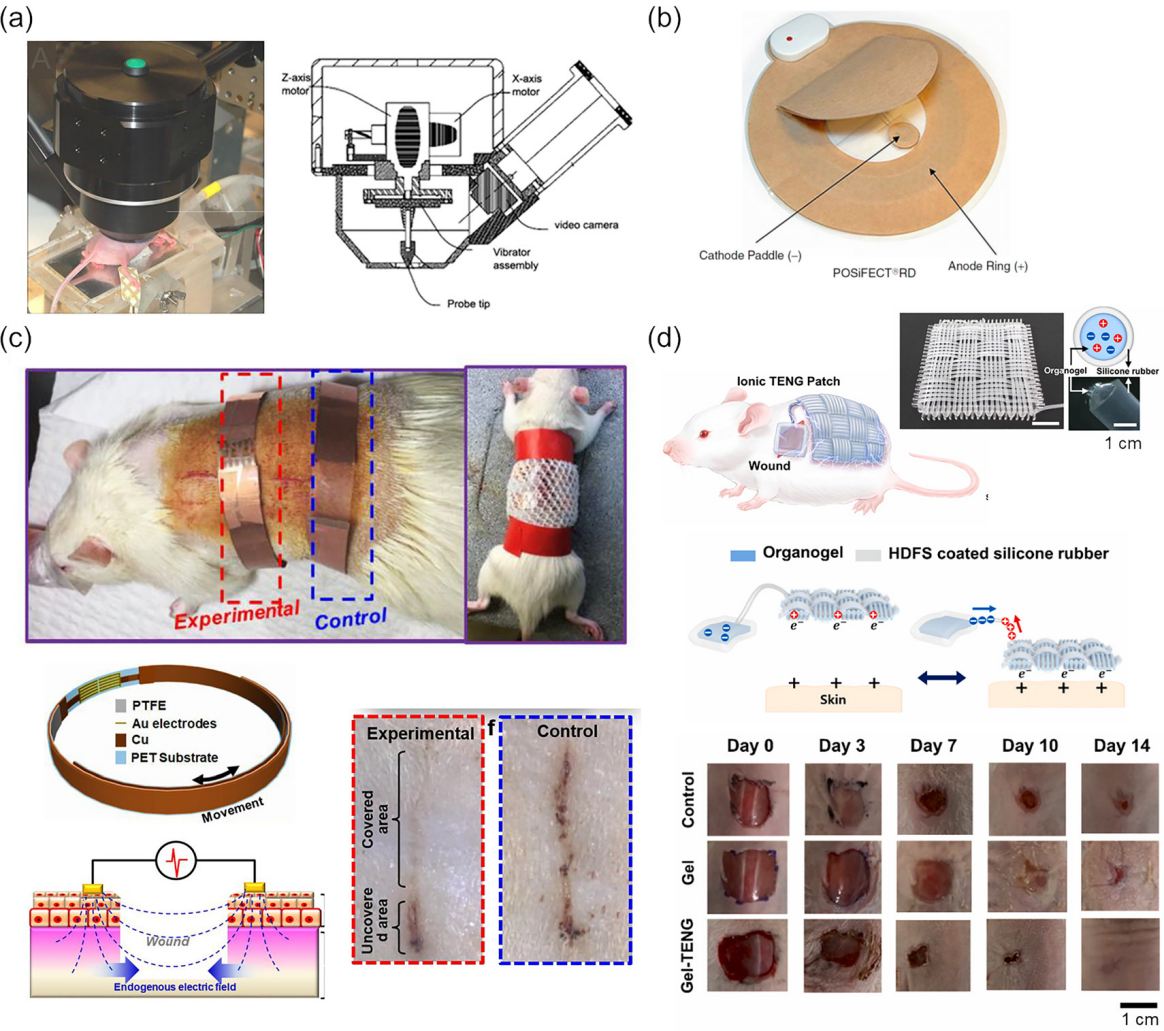
Devices for monitoring and promoting wound healing. (a) A device for skin surface potential evaluation (Dermacorder). The vibrating probe scanned the lateral potential distribution around the wound. Reproduced with permission from Nuccitelli *et al.*, Wound Repair Regener. **19**, 5 (2011).^235^ Copyright 2011 John Wiley and Sons. (b) A wireless electrical dressing powered by a disposable battery (POSiFECT^®^). Electrical current is applied through the hydrogel that covers the wound. From Wollina *et al.*, in *Measurements in Wound Healing*, edited by Mani *et al.*^237^ Copyright 2012 Springer-Verlag London. Reproduced with permission from Springer-Verlag London. (c) A wearable device with a piezoelectric nanogenerator (PENG). Wound closure and tissue regeneration were accelerated by pulsed electrical stimulation generated by the deformation of the device. Adapted with permission from Long *et al.*, ACS Nano **12**, 12 (2018).^241^ Copyright 2018 American Chemical Society. (d) A wearable device with a triboelectric nanogenerator (TENG). Wound closure and tissue regeneration were accelerated by pulsed electrical stimulation harvested from the friction between the TENG and the skin surface. Adapted with permission from Jeong *et al.*, Nano Energy **79**, 105463 (2021).^242^ Copyright 2020 Elsevier Ltd.

As mentioned above, local TEP decreases as the integrity of the tissue is impaired and fluctuates throughout the process of recovery.^140,146^ This indicates that the TEP value can be an electrical indicator for wound monitoring. In these early studies, salt bridges were placed in direct contact with the wounds to measure the potential difference and its regional variation. For clinical applications, a device with a vibrating probe was developed to determine the skin surface potential via capacitive coupling without direct contact [Fig. 8(a)].^156,235^ The lateral potential distribution was scanned around the wound on mouse and human skin, and the potential of the wounded area was found to be more negative than for the surrounding intact skin. Although the device was once in the market under the name of Dermacorder, the trademark expired in 2019 and the device is currently unavailable.

For the promotion of wound healing, a variety of electrical stimulation devices that enhance the therapeutic effect of the endogenous electrical current generated in the wound have been developed and are in clinical use. WoundEL^®^ system consists of a dressing electrode and a desktop-typed pulse generator designed to provide low-frequency, single-phase pulse stimulation. In a clinical study, accelerated wound healing and improved pain scores were reported.^236^ POSiFECT^®^ is an example of a wireless device with a disposable coin-cell battery, which delivers microcurrent through concentric electrodes embedded in a hydrogel dressing [Fig. 8(b)]. An effect on the initiation of healing of chronic wounds was observed, and the number of home clinician visits was decreased.^237^ In particular, a chronic wound imposes a burden on patients and the health care systems, and the development of a highly effective device that enables patient-centered treatment will continue to draw attention.

With evolving technologies related to wearable devices, self-powered bandages without large-sized power supplies have been developed. In 2017, Kai *et al.* developed a bioelectric plaster with a patch-typed biofuel cell made of organic materials.^238^ The flexible biofuel cell consisted of two enzymatic electrodes made of carbon fiber fabric coated with carbon nanotubes and functionalized with redox enzymes, and the reactive materials in it were biosafe species, fructose and oxygen. DC electrical stimulation generated across the wound restored the thick epidermal tissue without contracture on mouse skin *in vivo*.

In the same year, Bhang *et al.* reported a piezoelectric device based on layers of aligned ZnO nanorods and polydimethylsiloxane (PDMS).^239^ As the patch covering the wound was bent, the piezoelectric potential appeared across the nanorods, resulting in pulsed electrical stimulation. *In vivo* experiments showed that the device promoted wound closure and epidermal regeneration and enhanced wound healing processes, including angiogenesis. Piezoelectric devices have been further improved in terms of biocompatibility, whereby Du *et al.* presented accelerated wound closure and tissue regeneration with a piezoelectric nanogenerator (PENG) device, which was composed of electrospun fiber of polyvinylidenefluoride mounted on a self-adhesive hydrogel substrate.^240^

A triboelectric nanogenerator (TENG), an energy harvesting technology to convert mechanical energy to electrical current, is also an emerging power source for wearable electronics. In 2018, Long *et al.* fabricated an electrical bandage based on TENG [Fig. 8(c)].^241^ The nanogenerator, which was composed of a polytetrafluoroethylene (PTFE) layer and a Cu layer separated by a polyethylene terephthalate (PET) substrate, supplied discrete current flow according to the deformation of the device by the dressing electrode placed across the wound. *In vivo* experiments on a rectangular wound on the rat skin demonstrated faster wound closure than the control. More recently, a totally flexible TENG device, which was composed of elastomers and organogel and harvesting the triboelectricity generated between the skin and the device, was reported, presenting improved conformability and biocompatibility [Fig. 8(d)].^242^

Efforts are also being made to increase the conductivity of the electrode materials where the wound dressing material alone is intended to amplify the effect of the endogenous current. There is an example of conductivity with electrically conductive particles. Noninvasive electrical stimulation has been used in the treatment of melanoma with direct metallic printed electrodes.^243^

There are many more studies on functional wound dressings. Considering *in vivo* studies after 2019, electrically conductive dressings containing graphene oxide (GO),^244^ polypyrrole,^245,246^ MXene,^247^ and liquid metal integrated with a microneedle patch^248^ succeeded in accelerating wound healing with external electrical stimulation. In addition, the application of conductive dressings carrying GO,^249–252^ carbon nanotubes,^253,254^ polypyrrole,^255^ and polyaniline^256^ to the skin has been reported to enhance wound healing *in vivo* in the absence of external stimulation, such as application of heat or antibiotics. Many conductive/semi-conductive materials have been reported to have antimicrobial activity through various mechanisms, including chemical release, electrostatic interactions and redox reactions,^257–261^ and there are also a number of studies on *in vivo* wound healing using a dressing containing GO,^262–264^ silver nanomaterials,^265–268^ zinc oxide nanomaterials,^269^ and metal organic frameworks.^270–272^ With respect to rather older related studies, other schemes, such as electrets of hydroxyapatite,^273,274^ were also reported to be effective as wound dressings. Such functional materials could be attractive options for electrical devices for wound healing, allowing a more effective approach, especially for refractory or infected wounds. The search for extremely bio-safe materials that enhance healing efficiency is ongoing.

### C. Transdermal drug delivery and extraction of biomolecules

A number of products for iontophoretic drug delivery have already been approved by the FDA and are commercially available for medical and cosmetic use. Wireless patches that can be filled with various solutions include IontoPatch^TM^ (IontoPatch) and ActivaPatch^®^ (North Coast Medical, Inc.), which deliver a prescribed period/intensity treatment. As yet, not an inconsiderable number of marketed devices have safety and cost issues, and further technological improvement is still needed. A detailed review of the commercially available devices and clinical trials can be found in Refs. 159 and 161.

**FIG. 9.**
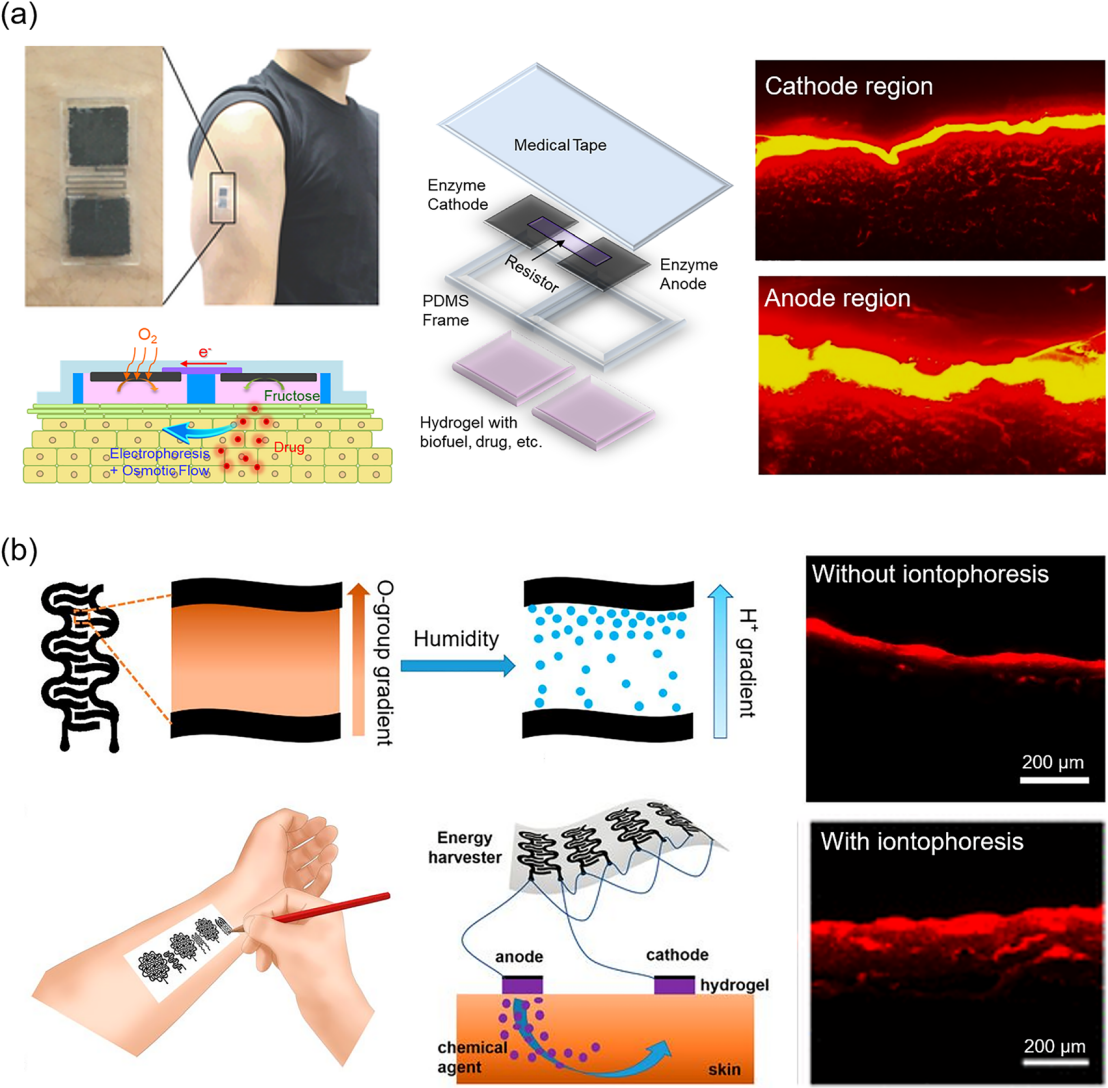
Wearable devices for transdermal drug delivery. (a) Totally organic iontophoresis patch. A biofuel cell based on the carbon fabric and conductive polymer was used as a power source to deliver the drug from the reservoir hydrogel into the skin. Iontophoresis of Rhodamine B at 50 *μ*A/cm^2^ for 1 h increased the penetration on the anode side. Adapted with permission from Ogawa *et al.*, Adv. Healthcare Mater. **4**, 4 (2015).^283^ Copyright 2014 John Wiley and Sons. (b) An iontophoresis patch powered by an energy harvester using pencil–paper electronics (graphite patterns drawn on a paper substrate). Graphene with a gradient of oxygen-containing groups adsorbs moisture, creating a gradient in the proton concentration produced by hydrolysis, which eventually diffuses. Eight units of harvester connected in parallel showed increased penetration of Rhodamine B at a relative humidity of about ca. 95%. Adapted with permission from Xu *et al.*, Proc. Natl. Acad. Sci. U. S. A. **117**, 31 (2020). Copyright 2020 National Academy of Sciences.^284^

Over the past few years, there has been a continued effort to develop advanced devices employing the latest technology. Choi *et al.* have demonstrated iontophoresis with thin graphene electrodes^275^ and devices physically adsorbed to the skin.^276^ With remarkable progress in power sources, the potential application of flexible lithium-ion batteries,^277^ TENG,^278,279^ and reverse electrodialysis batteries^280–282^ to power on-skin iontophoretic devices have been reported. In an effort to improve their biological safety, Ogawa *et al.* have developed an entirely organic iontophoresis patch consisting of carbon fabric and conductive polymer that was integrated with a biofuel cell [Fig. 9(a)].^283^ Xu *et al.* demonstrated iontophoresis driven by an energy harvester based on pencil–paper electronics (graphite patterns drawn on a paper substrate), which generates electricity in response to humidity [Fig. 9(b)].^284^ As a comprehensive system for mobile-controlled drug delivery, Reddy *et al.* reported a dual-channel iontophoresis device with a contact failure-detecting module.^285^

As mentioned above, iontophoresis can be combined with other schemes to enhance delivery. Kusama *et al.* demonstrated this concept using the porous microneedle modified with charged hydrogels.^286^ The device was integrated as a flexible patch-type device and powered by a biocompatible fuel cell. This scheme has evolved to directly bond charged polymers to the surface of pores to expand the potential applications in large molecules such as vaccines.^287^ Li *et al.* successfully transported charged insulin vesicles through porous microneedles, indicating the possibility of glycemic control.^288^

There are two methods for the extraction of biomolecules by iontophoresis: one is to deliver a sweat-inducing agent (e.g., pilocarpine) into the skin and collect the biomarkers in the secreted sweat. The other method is to collect the interstitial fluid extracted in the electroosmotic flow (reverse iontophoresis). For the former, the Macroduct^®^ and Nanoduct^®^ Sweat Analysis Systems (ELITechGroup, Inc.), which consist of a portable sweat inducer and disk electrodes, are approved by the FDA for laboratory diagnosis.^289,290^ Regarding reverse iontophoresis, Glucowatch^®^ Biographer (Cygnus, Inc.) was previously available on the market for glucose monitoring. Although it was later withdrawn, there is still considerable interest in transdermal monitoring, and the problems that were raised, such as skin irritation and the need of calibration, are being overcome in subsequent research.

**FIG. 10.**
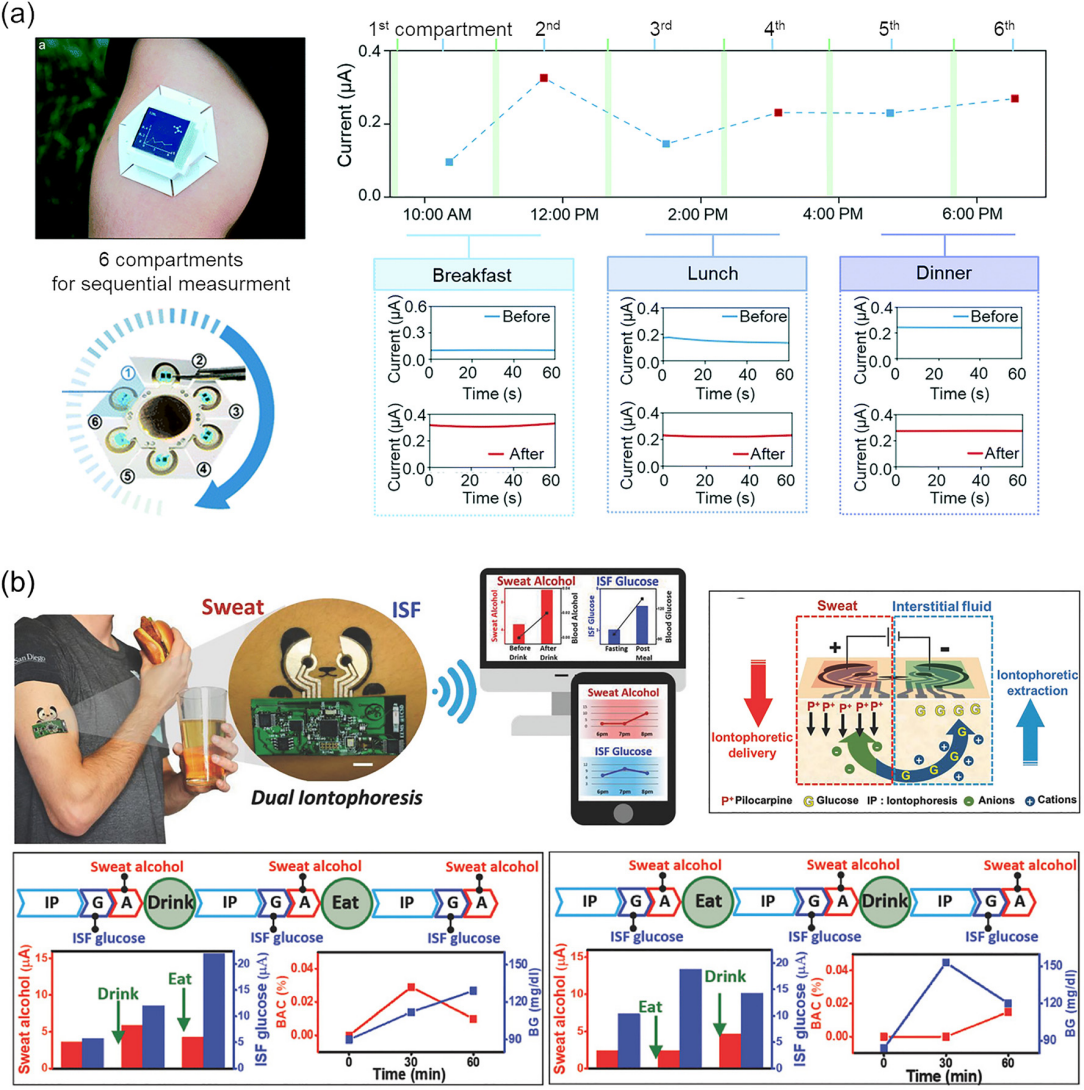
Wearable devices for transdermal extraction of biomolecules. (a) A wearable device with multiple compartments for tracking diurnal variations of biomarkers. The iontophoresis units in each compartment were activated one by one in a sequence to monitor changes in the sweat glucose concentration throughout the day. Adapted with permission from Hojaiji *et al.*, Lab Chip **20**, 24 (2020).^294^ Copyright 2020 Royal Society of Chemistry. (b) A multimodal sensor that performs iontophoresis of sweat-inducing agents and reverse-iontophoresis of interstitial fluids (ISFs). ISF glucose and sweat alcohol changed according to meal and alcohol intake, following the same trend as blood glucose (BG) and blood alcohol concentration (BAC). Adapted with permission from Kim *et al.*, Adv. Sci. **5**(10), 1800880 (2018).^292^ Copyright 2018 Author(s), licensed under a Creative Commons Attribution (CC BY) license.

To cite studies on devices from 2015 onwards, monitoring of alcohol,^291,292^ glucose,^292–294^ levodopa,^295^ and vitamin C^296^ from sweat samples collected by the pilocarpine delivery method has been reported. Simmers *et al.* succeeded in efficient and prolonged stimulation of perspiration by using carbachol instead of pilocarpine.^297^ Hojaiji *et al.* divided a wearable device into multiple detection units to monitor the time course of the glucose level [Fig. 10(a)].^294^ The graphs in Fig. 10(a) correspond to sweat glucose profiles during the day and real-time amperometric recordings at each period, showing that glucose levels elevated after the three main meals. Many of the reported devices were wearable, and the data were transported to external systems through wireless communication. Technological improvement has also been made in reverse iontophoresis. Bandodkar *et al.* demonstrated glucose monitoring at low current densities using on-skin (tattoo-based) electrodes with improved electrical contacts.^298^ Chen *et al.* reported an ultrathin biosensor system that promotes the glucose extraction into the ISF by the iontophoresis of hyaluronic acid, in parallel with the extraction by reverse iontophoresis.^299^ Lipani *et al.* developed a pixel array of glucose extraction/detection units to quantize the sampling from the follicular pathways, which is unevenly distributed on the skin surface.^300^ Sweilam *et al.* reported a textile based system for monitoring of lithium ion levels.^301^ Kim *et al.* developed a multichannel device that performs pilocarpine iontophoresis for sweat sampling and reverse iontophoresis for ISF sampling simultaneously [Fig. 10(b)].^292^ The graphs in Fig. 10(b) correspond to measured sweat alcohol, ISF glucose, blood glucose (BG), and blood alcohol concentration (BAC) before and after the intakes of meals and alcoholic beverages (with smaller amounts of sugar than the meals). The device showed increased glucose and alcohol signals after eating and drinking, consistent with trends in blood levels. Yang *et al.* promoted the collection of DNA via minimally invasive microneedles by reverse iontophoresis.^302^ With further advances in related technology, more biomarkers are expected to become available in multimodal devices as healthcare platforms for everyday life.

## IV. CHALLENGES AND OUTLOOK

The electrical properties of skin outlined in this review are promising research topics with potential applications in medicine and cosmetics as well as in basic science. Although the number of related methods and products established for use in practice is increasing as expected, there have been few attempts to integrate the underlying mechanisms that link those aspects in common, and some issues remain unresolved. One of the interesting aspects is that the skin is sensitive to changes in the external environment, making accurate characterization difficult, and advances in measurement and control techniques are required. An overview of both the passive and active electrical properties will provide a practical understanding and advance the maturity of the field. First of all, while the physical properties of macroscopic skin have been well studied, there is still little discussion that takes into account the nonuniformity of the microstructure. A better understanding of the regional difference of the electrical characteristics is essential for diagnostics with higher resolution and for treatment with current stimulation. As for the ionic signals in the epidermis, the microscopic dynamics are still controversial, and further research efforts are necessary for the practical application of ionic manipulation approaches. Technological advances in electrochemistry, including miniaturization of biocompatible electrodes for higher resolution mapping, may facilitate the measurement of precise electrical profiles. The identification of TEP carriers will also help determine efficient stimulation modalities for the regulation of tissue regeneration. Since physiological processes in viable skin may affect iontophoretic delivery, evaluation in experimental setups emulating living systems is essential to ensure the safety of voltage application and to expand the range of applicable chemicals. In addition, the analogy between the epithelial tissue lining the skin appendages and the epidermis may shed light on the efficacy and mechanism of action of appendage stimulation. The widespread diffusion of wearable/portable devices will introduce more biometric monitoring into everyday lives. In particular, rapid advances in e-skin related technologies may lead to convenient tracking by multimodal monolithic devices upon the overcoming of the current challenges in signal processing and integration. The growing interest in this area suggest that the electrical approaches to the skin will continue to evolve and contribute to significant developments in the medical and cosmetic fields.

## Data Availability

Data sharing is not applicable to this article as no new data were created or analyzed in this study.
